# AI Analysis of General Medicine in Japan: Present and Future Considerations

**DOI:** 10.2196/52566

**Published:** 2024-03-29

**Authors:** Nozomi Aoki, Taiju Miyagami, Mizue Saita, Toshio Naito

**Affiliations:** 1 Department of General Medicine Juntendo University Faculty of Medicine Tokyo Japan

**Keywords:** artificial intelligence, physicians, hospitalists, polypharmacy, sexism, Japan, AI, artificial intelligence, medicine, Japan, gender-biased, physicians, physician, medical care, gender, polypharmacy, women, Pharmacology, older adults, geriatric, elderly, Japanese

## Abstract

This paper presents an interpretation of artificial intelligence (AI)–generated depictions of the present and future of general medicine in Japan. Using text inputs, the AI tool generated fictitious images based on neural network analyses. We believe that our study makes a significant contribution to the literature because the direction of general medicine in Japan has long been unclear, despite constant discussion. Our AI analysis shows that Japanese medicine is currently plagued by issues with polypharmacy, likely because of the aging patient population. Additionally, the analysis indicated a distressed female physician and evoked a sense of anxiety about the future of female physicians. It discusses whether the ability to encourage the success of female physicians is a turning point for the future of medicine in Japan.

## Introduction

The concept of “general medicine in Japan,” born around the 1970s, led to 2 main physician categories in Japan: family doctors and hospitalists. Unlike general medicine departments overseas, there are no barriers to practicing within either family medicine or hospital medicine; both are unique specialties that require the adaptation of work to the size and conditions of the institutions they belong to, such as hospitals and clinics [1]. Additionally, some general medicine department managers are physicians who have transferred from specialized departments, and the mindset and competencies differ across institutions. Thus, the state of general medicine in Japan is unclear, and we have been unable to provide a clear answer despite more than 50 years of unremitting discussion. Since then, the field of general medicine has gradually expanded, and as of 2017, 86% of university hospitals have a department of general medicine [2]. Although the Japanese government and patients themselves have recently begun to recognize the importance of general medicine doctors, the public still lacks awareness of general medicine practitioners who do not require patients or their families to visit the hospital [3,4]. Therefore, we used artificial intelligence (AI) to assess the society’s perception of general medicine doctors in Japan. What answer will AI provide?

## What Is the Answer Shown by AI ?

We used a tool called DALL-E (Open AI), which analyzes text from a neural network and produces a graphical representation. We inputted the text “General medicine in Japan” and “Future of general medicine in Japan” to generate fictitious images [5].

### General Medicine in Japan

**Figure 1:**
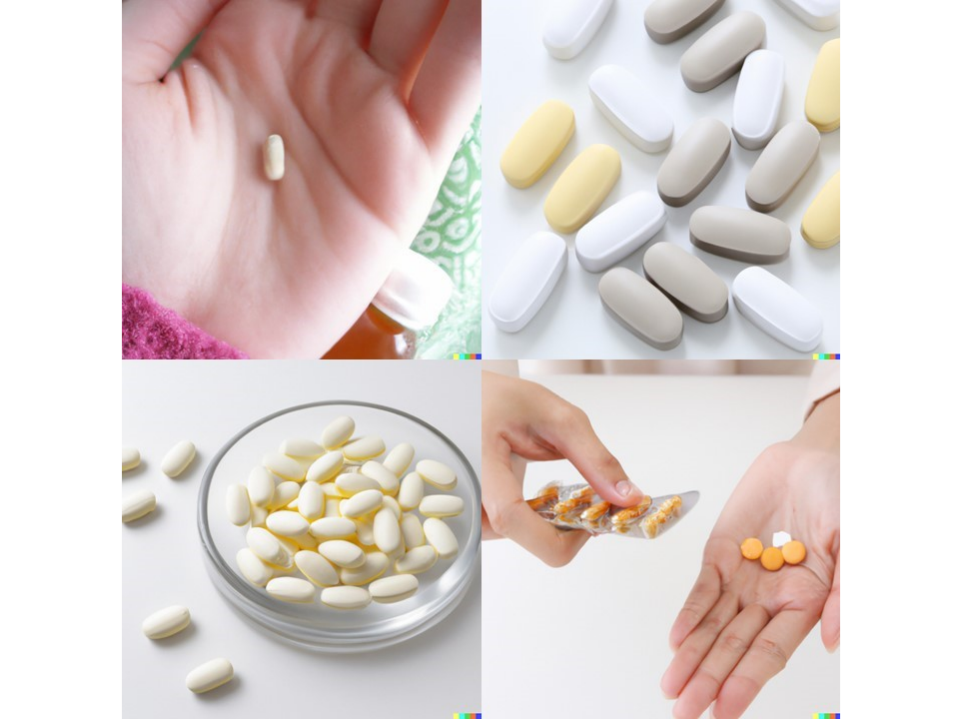
General medicine in Japan.

The entry “General medicine in Japan” generated a picture of several drugs (Figure 1), which we interpreted as being indicative of the polypharmacy observed in Japanese medicine. Compared to the rest of the world, Japan is a superaging society. As of 2013, 25% of the population is older than 65 years [6]. Polypharmacy, the combination of various drug therapies for comorbid diseases, is one of the problems seen within an aging society [7]. In Japan, polypharmacy has other causes. For example, all citizens are covered by medical insurance, providing ease of access to hospitals and medical care at reasonable fees at any time. One of the disadvantages of this system is that patients can see specialists immediately, which is why family doctors have fewer patients. This further encourages polypharmacy as patients visit multiple departments [8], leading to a growing need for doctors who can provide broad-spectrum diagnostic and treatment opportunities to patients. Both general doctors with diverse work backgrounds and polypharmacy are complex issues in Japan, which require total reform. Based on this concept, AI may have created polypharmacy as a graphic representation of the most common medical issue seen in Japan.

### Future of General Medicine in Japan

**Figure 2:**
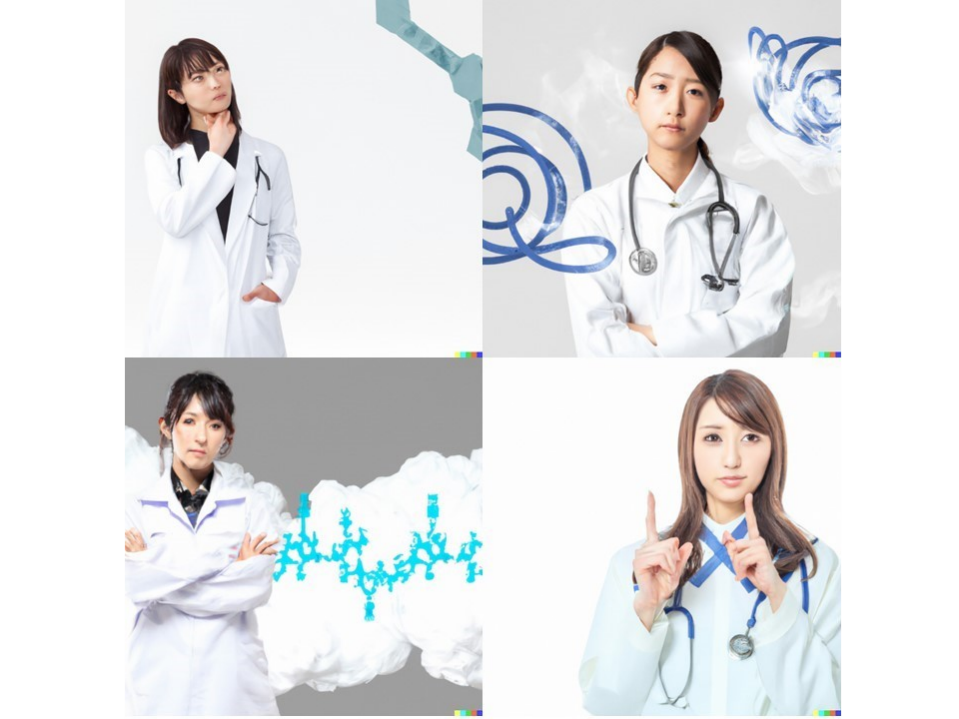
Future of general medicine in Japan.

Interestingly, “Future of general medicine in Japan” presented a completely different graphic. Despite approximately 70% of doctors in Japan being male, only 4 female doctors were depicted to be distressed (Figure 2). Although the percentage of female doctors in Japan is on the rise, in 2018, it was reported that women are less likely to be accepted into some medical schools than men [9], therefore raising awareness of sex discrimination in the Japanese medical community. Several reports describe the difficulties of working as a female doctor; during promotions and evaluations, women in Japan often feel discriminated against [10-13]. Moreover, the gender gap at work in Japan is by far the largest among high-income countries [14]. Currently, all the 19 medical specialty societies in Japan are primarily represented by men; a survey study of the actual state of general medicine found that the ratio of men to women was 9:1, indicating that women are grossly underrepresented in general medicine [15]. Many within general medicine have expressed concern about the impact of this issue on future health care. In response, the Japanese Society of Hospitalists and General Medicine established a division for female physicians in February 2021. In 2022, as part of the Women Physicians Subcommittee’s activities, national and international comparisons of female hospitalists were proposed [16]. However, activity still remains limited: the inclusion of women has increased only slightly since inception, and the number of female leaders remains low [16]. It must also be stated that an absence of women in leadership positions can cause female doctors to lose their role models, create an even wider gender gap, and encourage further bias in the medical workforce [17].

The future that AI envisioned may have focused on female physicians, who are currently reported to be oppressed in Japan. As a solution, it is necessary to support the activities of female physicians in general medicine and develop a workplace where women can work comfortably as general physicians. It has been reported that older hospitalized patients treated by female internists have lower mortality and readmission rates than those cared for by male internists [18]. The activities of female physicians may have important clinical significance for future medical care in the superaged Japanese society. Social change in the current gender-biased image of physicians and acceptance by the population is a great hope for Japanese medicine.

### Limitations

Using AI, we assessed the current and future status of general medicine practice in Japan. However, the criteria used to select the AI image output are unclear, making the academic evidence less credible. Scientific publications on the subject of AI have been submitted since 2023. While it has been reported that AI may be an effective tool in medical education [19], careful evaluation of the preparation of the article is recommended [20]. However, the picture painted by the AI in this report is consistent with previously reported social facts. It is a perspective that we humans are unaware of or avoid looking at; we believe that this view cannot be easily ignored.

### Conclusions

AI depicts “General medicine in Japan” as polypharmacy, the “Future of general medicine in Japan” as distressed female physicians. While this AI-generated picture cannot be verified or avoided, it shows the future challenges associated with the practice of general medicine in Japan.

## Abbreviations

AI: artificial intelligence
